# Clinical Deterioration in Dogs with Idiopathic Epilepsy Caused by *E. coli* Urinary Tract Infection

**DOI:** 10.3390/ani15172562

**Published:** 2025-08-31

**Authors:** Solveig Reeh, Teresa Schmidt, Holger Andreas Volk, Nina Meyerhoff

**Affiliations:** 1 Department of Small Animal Medicine and Surgery, University of Veterinary Medicine Hannover, 3559 Hannover, Germany; 2 Gastrointestinal Laboratory, School of Veterinary Medicine and Biomedical Sciences, Texas A&M University, College Station, TX 77840, USA

**Keywords:** idiopathic epilepsy, anti-seizure medication, urinary tract infection, *E. coli*

## Abstract

Idiopathic epilepsy (IE) is a common chronic neurological disorder in dogs, and both its comorbidities and adverse effects of anti-seizure medication (ASM) can markedly reduce quality of life (QoL) for affected dogs and their caregivers. Concurrent conditions such as lower urinary tract infections (UTIs) may mimic ASM side effects or signs of disease progression, potentially leading to inappropriate dose adjustment or treatment discontinuation. This retrospective case series describes eight dogs with IE and *Escherichia coli* (*E. coli*) UTI, presenting with suspected worsening of ASM side effects. Reported deterioration lasted 1–55 days (mean 31), with behavioral changes (n = 5), lethargy (n = 5), new or worsened ataxia (n = 5), urinary incontinence (n = 3), polyuria (n = 3), polydipsia (n = 2), and additional signs such as weakness, exercise intolerance, panting, and cluster seizures. All dogs showed bacteriuria; urinary specific gravity was 1.020 ± 0.013 (mean ± standard deviation) [range; 1.002–1.042]. *E. coli* (>10^6^ CFU/mL) was isolated in all cases. Treatment with amoxicillin–clavulanic acid resulted in clinical improvement within 24–72 h; five dogs experienced UTI relapse, again with clinical deterioration. Findings emphasize the importance of recognizing and treating UTIs in epileptic dogs to avoid misinterpretation as ASM toxicity and possible worsening of seizure control. Prospective studies are needed to clarify potential links between ASM, urinary dilution, immune modulation, and infection risk.

## Simple Summary

This case series describes eight dogs with idiopathic epilepsy (IE) that were diagnosed with concurrent urinary tract infections (UTIs) caused by *Escherichia coli*. Clinical signs of UTIs—such as lethargy, polyuria, and polydipsia—can mimic or exacerbate adverse effects of long-term anti-seizure medication (ASM). In the cases described here, UTIs appeared to aggravate ASM side effects and impair seizure control. Clinical findings, diagnostic test results, treatments, and follow-up outcomes are presented. The potential mechanisms are discussed, including phenobarbital-associated effects on the urinary tract that may predispose dogs to UTIs, and infection-related inflammatory processes that could trigger epileptic seizures. These cases highlight the importance of considering UTIs as a comorbidity in dogs with epilepsy and of performing regular urinalysis during ASM therapy. Early recognition and appropriate treatment of UTIs may prevent unnecessary changes in ASM regimens, avoid prolonged clinical deterioration, and improve the quality of life for affected dogs and their caregivers.

## 1. Introduction

Idiopathic epilepsy (IE) is one of the most common chronic neurological conditions in dogs, with an estimated prevalence of 0.6–0.75% in the general canine population and up to 5% in certain breeds [1,2]. As a lifelong disease, IE poses a significant burden not only on affected dogs but also on their caregivers. Seizures themselves, comorbid behavioral abnormalities, and the long-term side effects of anti-seizure medication (ASM) all contribute to a reduction in quality of life (QoL) for both dogs and owners [3,4]. In some cases, the strain may lead to premature euthanasia, while in others, concern over medication side effects may prompt dose reductions or discontinuation, thereby increasing the risk of seizure recurrence [5].

Phenobarbital remains a widely used first-line ASM in dogs, but its administration is frequently associated with side effects such as sedation, ataxia, polyphagia, polydipsia, and polyuria [6,7,8]. The changes in fluid intake and urine output can lead to persistently decreased urine specific gravity (USG), potentially compromising the urinary tract’s defense mechanisms and increasing the risk for bacterial infection [9,10,11]. Recent veterinary studies have shown that dogs with persistently diluted urine—particularly those with isosthenuria—have a significantly increased risk of bacteriuria, most commonly caused by *E. coli* [9]. In one study of dogs with chronic kidney disease, those with USG < 1.012 were more than twice as likely to yield positive urine cultures [11]. Proposed mechanisms for increased bacterial growth in dilute urine include a reduction in natural antimicrobial factors such as urea and salts, decreased mechanical flushing due to lower urinary concentration, and reduced osmotic stress on the bacteria [9].

Although these infections are typically regarded as localized and uncomplicated, their systemic effects may extend beyond the urinary tract. In human medicine, seizure activity has been documented in infants with acute pyelonephritis [12]. Similarly, cases of bacteriuria-associated encephalopathy and new onset seizures have been reported in dogs and cats, with clinical signs resolving rapidly following antimicrobial treatment [13]. Notably, in a recent study by our group investigating novel therapy for epilepsy [14], some dogs were excluded due to concomitant urinary tract infections with *E. coli*. In these instances, the infection was presumed to exacerbate seizure activity, representing a possible cause of pseudoresistance [15]. Recognition of UTIs in this context is further complicated by the fact that polyuria and polydipsia—common side effects of standard ASM—can mask the clinical signs of infection. The present study aims to describe these cases in order to raise awareness of urinary tract infections as a potential comorbidity that may worsen the course of canine epilepsy and its management.

## 2. Materials and Methods

Digital medical records from the Neurology Department of the Small Animal Clinic at the University of Veterinary Medicine Hannover (TiHo) were reviewed for the years 2020 to 2023. The search focused on dogs diagnosed with idiopathic epilepsy and a concurrent UTI caused by *E. coli*.

Dogs were eligible for inclusion if they had a complete medical record and fulfilled diagnostic criteria for idiopathic epilepsy according to the International Veterinary Epilepsy Task Force (IVETF) Tier I or II. Tier I diagnosis requires a seizure onset between the ages of 6 months and 6 years, unremarkable interictal neurological examination, and normal laboratory tests. Tier II diagnosis includes all Tier I criteria plus unremarkable magnetic resonance imaging (MRI) of the brain and cerebrospinal fluid (CSF) analysis to exclude other causes of seizures [16] In addition, the presence of clinical signs consistent with UTI and confirmatory findings in urine analysis and microbiological culture from sterilely collected samples (via cystocentesis) indicating *E. coli* infection were required. Dogs were excluded from the study if their medical records were incomplete, if the UTI had occurred prior to the diagnosis of idiopathic epilepsy, if no *E. coli* was detected in the urine culture, or if the clinical condition at the time of presentation was significantly influenced by other acute systemic diseases.

Data recorded included signalment, medical history, clinical signs, results of general and neurological examination, imaging, ASM and antibiotic therapy, as well as treatment response and outcome. At the time of UTI diagnosis, serum concentrations of ASM were routinely measured. In addition, available data on ASM dosages and serum concentrations from routine therapeutic drug monitoring before and after the UTI episode were collected and included in the analysis. To evaluate whether phenobarbital dosages or serum concentrations differed before versus during UTI, values from paired time points were compared. Data distribution was assessed for normality (Shapiro–Wilk test), and paired *t*-tests were applied accordingly. Post-UTI values were not included in this analysis, as they were not consistently available across all cases, and in some dogs, phenobarbital dosages were later increased due to seizure progression (Supplementary Table S2).

Urine was collected via ultrasound-guided cystocentesis for standard urinalysis, including quantitative measurement of urine specific gravity using a refractometer, qualitative dipstick testing for blood, nitrite, glucose, and ketones, and qualitative cytological sediment examination. Samples were also submitted for microbiological culture. All dogs received an abdominal ultrasound—either of the entire abdomen or focused on the urogenital tract. Clinical signs were evaluated in relation to laboratory findings.

## 3. Results

### 3.1. Study Population

Eight client-owned dogs diagnosed with idiopathic epilepsy and *E. coli* lower UTI were included. The cohort consisted of a variety of medium to large breed dogs (mean body weight: 36.4 kg; range: 20.4–57.4 kg), including two Border Collie mixes, and one each of Saint Bernard, Grand Basset Griffon Vendéen, Australian Shepherd, Australian Doodle, Kangal, and Greater Swiss Mountain Dog. Two dogs were intact males, three were intact females, and three were spayed females. The age at diagnosis of UTI ranged from 2.5 to 7.5 years (mean 4.8 years).

### 3.2. Epilepsy History and Treatment

All dogs had previously been diagnosed with idiopathic epilepsy. Seven dogs fulfilled IVETF Tier II criteria; one dog was classified as Tier I with an unremarkable contrast-enhanced CT of the head. All dogs had chronic recurring generalized tonic-clonic seizures and were undergoing chronic anti-seizure treatment with phenobarbital at the time of presentation. Four dogs received potassium bromide as adjunctive therapy, which had been administered chronically for a period of 9 months up to 1 year, and one dog also received imepitoin in combination. Supportive measures included dietary supplementation with medium-chain triglycerides in three dogs. The time between epilepsy diagnosis and UTI onset ranged from 2 to 51 months (median 29.5 months). Phenobarbital dosages ranged from 2.2–6.3 mg/kg BID (mean 3.6 mg/kg), with serum concentrations between 16.0–43.7 µg/mL (mean 28.1 µg/mL). Potassium bromide concentrations (n = 4) were within the therapeutic range (955–2104 mg/L, mean 1328 mg/L).

**Figure 1:**
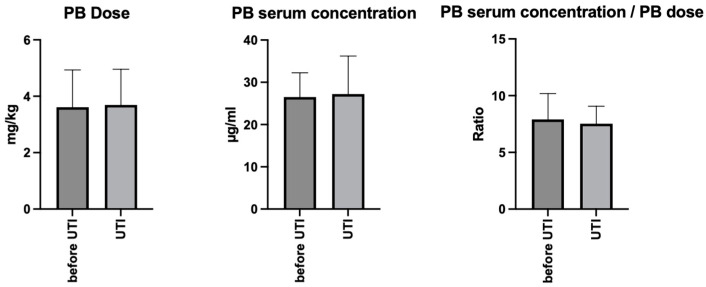
Phenobarbital dosages (mg/kg BID), serum concentrations (µg/mL), and the ratio of serum concentration to dosage in eight dogs with idiopathic epilepsy before and when urinary tract infection (UTI) occurred (*p* > 0.05, paired *t*-test for all comparisons).

Statistical comparison of phenobarbital dosage and serum concentrations before versus when the dogs developed the UTI revealed no significant differences (paired *t*-test, *p* = 0.35 for dosage, *p* = 0.80 for serum concentrations). The ratio of serum concentration to dosage likewise showed no significant difference (*p* = 0.53) (Figure 1).

### 3.3. Clinical Signs and Initial Deterioration

**Figure 2:**
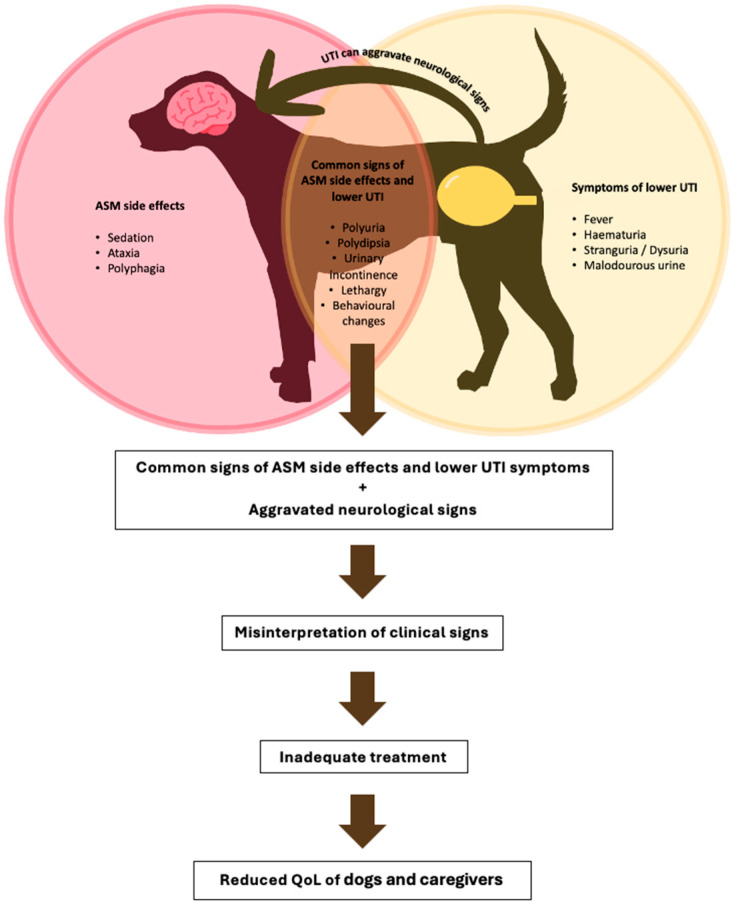
Overlap of clinical signs in idiopathic epileptic dogs with antiseizure medication (ASM) side effects and lower urinary tract infection (UTI) and the effect on the quality of life (QoL) of dogs and caregivers.

Prior to presentation, all dogs had shown signs commonly attributed to ASM side effects, including mild ataxia, polyuria, polydipsia, behavioral changes, and polyphagia. In two spayed female dogs, chronic urinary incontinence had been noted prior to the UTI episode. A gradual worsening of clinical signs over 2 weeks to 2 months led to presentation at the hospital for reassessment. At presentation, the following signs aggravating the neurological conditions were documented: behavioral changes (restlessness, n = 5; increased anxiety, n = 2), worsened sedation (n = 5), new onset or worsened ataxia (n = 5), as well as exercise intolerance (n = 1), tetraparesis (n = 1). One dog depicted additionally ambulatory weakness and tetraparesis, generalized reduced muscle tone, and decreased flexor reflexes in all limbs at presentation. Additionally, one dog presented with new onset cluster seizures. The more classical clinical signs associated with UTI were polyuria (n = 3), urinary incontinence (n = 3), polydipsia (n = 2), panting (n = 1), (n = 1), unpleasant scent (n = 1), and overgrooming of the anogenital region (n = 1), and two dogs showed elevated body temperature at presentation (Figure 2).

### 3.4. Diagnostic Findings

Complete blood count, clinical serum biochemistry, electrolytes, and bile acids were performed in all dogs. Mild laboratory abnormalities included eosinophilia (n = 1), borderline neutropenia (n = 1), mild leukopenia and thrombocytopenia (n = 1), leukocytosis and neutrophilia (n = 1), and mild elevation of liver enzyme activity (n = 2).

Urinalysis revealed consistent findings across all cases, including hematuria, pyuria, and bacteriuria. Three dogs had a positive nitrite test, and two exhibited mild proteinuria. Cytology in one case revealed dysplastic epithelial cells. Urinary specific gravity was 1.020 ± 0.013 (mean ± standard deviation) [range: 1.002–1.042]. In all dogs, *E. coli* was isolated in concentrations >10^6^ CFU/mL. An abdominal ultrasound was performed in four dogs, while in another four dogs, specifically the urogenital tract was examined. Prostatic enlargement was detected in one dog, and suspected chronic kidney disease was noted in another. All dogs were diagnosed with *E. coli* UTI based on microbiology and clinical signs.

### 3.5. Treatment and Response

All dogs received oral amoxicillin–clavulanic acid at dosages ranging from 12.3 mg/kg to 19 mg/kg twice daily (mean 14.6 mg/kg BID) for 7 to 16 days (mean 11 days). Clinical signs markedly improved in all dogs within 24–72 h of initiating antimicrobial therapy. The dog with new onset cluster seizures had no further cluster seizures at follow-up (37 days).

To evaluate treatment success, follow-up urinalysis was performed in all dogs between 7 and 16 days after initiating antibiotic therapy (mean: 11 days). In one dog, cytological examination revealed mild leukocyturia. In all other dogs, follow-up urinalysis showed no evidence of leukocytes or bacteriuria. Additionally, microbiological culture was performed in three dogs—including the dog with mild leukocyturia—and yielded negative results in all cases. Five dogs experienced at least one recurrence of *E. coli* UTI during the follow-up period (10–973 days). In one of these dogs, five relapses occurred within several months. Notably, this dog showed repeated pre-relapse deterioration of ataxia and lethargy in each episode. In all relapse cases, *E. coli* was again identified as the causative pathogen. The isolates were non-multidrug-resistant, and repeat antibiotic treatment was successfully conducted with amoxicillin–clavulanic acid. One dog had no recurrence, and in two dogs, recurrence status could not be assessed due to limited follow-up. In dogs with recurrent urinary tract infections, additional supportive measures were implemented alongside antibiotic therapy. These included peri-anal hygiene using chlorhexidine shampoo in one dog and administration of the nutraceutical Vesica^®^ (Inuvet GmbH, Lörrach, Germany)—containing cranberry extract, D-mannose, and N-acetyl-D-glucosamine—in another.

In addition, as shown in Supplementary Table S1, PB serum concentrations were recorded in six of the eight dogs 3–28 weeks after the UTI appointment. An increase in PB dosage was implemented in two dogs. At the follow-up appointment, after the PB dosage was increased and a corresponding rise in serum PB concentration was observed in the two dogs, the clinical signs previously noted during the UTI episode were no longer present in any of the dogs. The most important results are presented in Table 1.

## 4. Discussion

### 4.1. Phenobarbital and Polydipsia as Risk Factors for Recurrent UTI

This case series underscores the significance of lower UTIs as a potential comorbidity that can exacerbate clinical signs in dogs with IE receiving long-term ASM therapy. In all eight cases presented, systemic and neurological deterioration—manifesting as worsening ataxia, behavioral changes, and an increase in seizure severity—preceded the diagnosis of *E. coli* UTI, with prompt clinical resolution following antimicrobial treatment. Notably, these clinical declines were frequently prolonged and initially misattributed to ASM side effects or the natural progression of epilepsy, delaying appropriate diagnosis and intervention. These findings highlight the need for heightened clinical vigilance for UTIs in this patient population, especially as polyuria and polydipsia—common ASM side effects—can obscure recognition of UTIs [6,7,8]. This overlap may lead to misinterpretation of infection-related clinical signs as adverse drug reactions, potentially prompting unnecessary changes in ASM protocols.

Some of the most common ASM side effects—such as restlessness, polyuria, sedation, and behavioral changes—are non-specific and may overlap with signs of other systemic conditions, including UTI. As a result, such infections may be overlooked, especially when clinical signs are interpreted solely in the context of ASM adverse effects or comorbid epilepsy-related behavior changes. This diagnostic ambiguity is clinically significant, as it may lead to inappropriate dose reduction, treatment discontinuation, or even euthanasia due to perceived treatment failure or adverse events, ultimately compromising seizure control and QoL for both patients and caregivers [4].

Importantly, five out of eight dogs experienced at least one relapse of *E. coli* UTI during follow-up, each time associated with renewed neurological deterioration. This raises the hypothesis that ASM—particularly phenobarbital, due to its known association with polydipsia—may indirectly contribute to urinary dilution, which could facilitate recurrent UTI in this patient population [6,7,8]. Low USG, observed mostly across cases, has been associated with increased bacterial growth and adherence in vitro, likely due to reduced antimicrobial activity, mechanical flushing, and osmotic stress [9,11]. While other studies suggest that highly concentrated urine may also predispose to UTI under certain conditions (e.g., acidic pH) [17,18], persistently diluted urine remains a plausible risk factor in epileptic dogs receiving phenobarbital.

### 4.2. Immunomodulatory Effects of Antiseizure Medications

In human medicine, certain ASMs such as topiramate and levetiracetam have been associated with a mildly increased infection risk [19], while retrospective analyses link phenytoin, valproate, and carbamazepine to higher UTI incidence [20]. The immunomodulatory effects of ASMs may involve alterations in cytokine production, T-cell signaling, and suppression of humoral immunity [21,22].

Although these effects are not well-studied in dogs, transient neutropenia and blood dyscrasias have been reported in phenobarbital-treated animals [23,24]. These hematological abnormalities are believed to result from reversible bone marrow suppression, as evidenced by hypoplastic bone marrow findings and recovery after drug withdrawal. While the exact mechanism remains unclear, both direct toxic effects and idiosyncratic immune-mediated reactions have been proposed. Although this does not conclusively prove immunosuppression, such bone marrow suppression may indicate a transient impairment of immune competence. In humans, deviations in immunoglobulin profiles have also been observed during phenobarbital treatment; however, immunoglobulin levels were not assessed in our cases.

### 4.3. Urinary Tract Infection as a Seizure Aggravating Factor

Systemic infections, such as UTI, may also aggravate seizure frequency via para-infectious mechanisms. These include proinflammatory cytokine cascades and neuroinflammation, both well-established contributors to seizure worsening in human epilepsy [25,26,27]. In one study, up to 52.8% of breakthrough seizures in pediatric patients were linked to systemic infections [28]. Experimental data support the role of inflammation in epileptogenesis and ictogenesis, yet this is an underexplored area in veterinary epilepsy. In our cohort, seizure control improved following treatment of the infection in several dogs, indicating that similar mechanisms may be at play.

The overlap between infection-related neurological signs and epilepsy is further highlighted by reports of reversible encephalopathy, forebrain signs, and even seizures associated with bacteriuria in both veterinary and human medicine [13,29]. In rare cases, severe immune-mediated conditions such as Guillain–Barré syndrome have been triggered by *E. coli* UTI [30,31], supporting the idea that bacterial infections can provoke a spectrum of neurological manifestations.

In the context of infection on seizure control, it is important to consider that seizure worsening in treated epileptic dogs may not always reflect true pharmacoresistance. Kajin et al. (2023) demonstrated that up to 27% of dogs classified as drug-resistant were in fact pseudoresistant due to factors such as subtherapeutic drug levels, misdiagnosis, or owner non-compliance. Based on our findings, undiagnosed comorbid infections such as UTIs may represent an additional, underrecognized cause of pseudoresistance [15].

### 4.4. Stability of ASM Dosages and Serum Concentrations

Some of the most common ASM side effects—such as restlessness, polyuria, sedation, and behavioral changes—are non-specific and may overlap with signs of systemic conditions, including urinary tract infection (UTI). In all eight dogs, phenobarbital dosages and serum concentrations did not differ significantly between measurements obtained before and during UTI, indicating that fluctuations in ASM exposure were not responsible for the observed deterioration. Post-UTI values were not consistently available across all cases; however, in two dogs, phenobarbital dosages were later increased due to progressive seizure frequency. Importantly, these adjustments were not associated with the recurrence of clinical signs resembling ASM adverse effects or with new UTI episodes, even if the PB serum concentration was higher in some dogs. All dogs receiving potassium bromide had been in stable treatment for 9 months to 1 year prior to UTI, with therapeutic serum concentrations confirming steady state.

Taken together, these findings suggest that the neurological deterioration observed at the time of UTI was not linked to ASM fluctuations but rather to the infection itself. This interpretation is supported by the consistent clinical improvement following antimicrobial treatment. However, the exact direction of causality remains unclear: UTIs may exacerbate existing ASM side effects, while ASM-associated polyuria and polydipsia may in turn predispose to recurrent UTIs. Both pathways are biologically plausible, but conclusive evidence is lacking. Importantly, the overlap between UTI-related signs and common ASM adverse effects means that UTIs can mimic drug adverse events or loss of seizure control and may therefore be overlooked in clinical practice. UTIs should thus be recognized as a potential confounding factor and an underdiagnosed comorbidity in dogs with idiopathic epilepsy, with important implications for the assessment of ASM tolerability and seizure control.

### 4.5. Clinical Relevance and Study Limitations

Finally, the distinction between clinically relevant and subclinical bacteriuria remains a matter of debate [32,33,34]. While asymptomatic bacteriuria may not warrant treatment in all cases, its presence in dogs with epilepsy and neurological decline should prompt further evaluation and potential treatment. In our series, all dogs improved after antibiotic therapy—supporting the notion that even mild bacteriuria may be clinically meaningful in this context [13]. Therefore, when dogs with IE present for a recheck, clinicians should first assess blood parameters and serum levels of the administered anti-seizure medications, followed by urinalysis as the next step.

This study has several limitations. As a retrospective case series, diagnostic work-up and follow-up intervals varied between patients, and long-term follow-up was not available for all dogs. Prospectively recorded data on seizure frequency during the peri-infectious period were lacking, making it difficult to definitively link UTI to seizure exacerbation. However, one dog showed a new onset of cluster seizures that were resolved following antimicrobial therapy. It cannot be ruled out that the antibiotic treatment may have incidentally addressed an undiagnosed condition (e.g., encephalitis); however, we consider this very unlikely based on the clinical presentation and improvement following antibiotic therapy.

The cases presented here represent a subset of a larger prospective cohort study on idiopathic epilepsy. Within this cohort, 8 out of 34 dogs (23.5%) were diagnosed with both idiopathic epilepsy and at least one urinary tract infection during the observation period. Although this relatively high proportion may suggest that urinary tract infection is an underrecognized comorbidity in dogs with epilepsy, the sample size is too limited to draw definitive conclusions about prevalence in the broader canine population. Nevertheless, this finding underscores the need for increased clinical awareness and supports the rationale for further prospective studies involving larger cohorts.

## 5. Conclusions

In dogs with IE, particularly those receiving phenobarbital, recurrent UTIs may contribute to clinical deterioration through exacerbation of ASM side effects and potentially impaired seizure control. We recommend routine urinalysis in all dogs with IE presenting at a recheck appointment and urine culture via cystocentesis in epileptic dogs presenting with sudden behavioral changes, worsening adverse effects, or seizure destabilization. Early detection and treatment of UTIs may help prevent unnecessary alterations in ASM regimens, improve quality of life, and reduce the risk of euthanasia due to misattributed side effects.

## Figures and Tables

**Table 1:** Clinical data of eight dogs with idiopathic epilepsy and concurrent *E. coli* urinary tract infection (UTI) (ASM: anti-seizure medication; PhB: phenobarbital; KBr: potassium bromide; USG: urine specific gravity; UTI: urinary tract infection; n/a: not available).

Case	Breed	Sex	Age (y)	Weight (kg)	PhB Level (µg/mL)	KBr Level (mg/L)	USG	Bacteriology	ASM/Epilepsy-Related Signs	UTI-Related Signs	Treatment & Outcome	Relapses
1	Border Collie x	fn	4.5	35.4	21.5	2104	1.002	*E. coli* 10^6^	Restlessness, lethargy, tetraparesis, weakness	Hyperthermia, polyuria	Improved within 48 h after amoxicillin-clavulanic acid	0
2	St Bernard	f	5.75	43.8	21.6	n/a	1.016	*E. coli* 10^6^	Lethargy, polydipsia	Urinary incontinence, malodorous urine, overgrooming	Improved within 48 h after amoxicillin-clavulanic acid	0
3	Grand Basset Griffon Vendéen	fn	6	24.0	43.7	n/a	1.018	*E. coli* 10^6^	Restlessness, anxiety, lethargy	Worsened urinary incontinence	Improved within 24 h after amoxicillin-clavulanic acid	1
4	Australian Shepherd	f	7.5	23.6	36.3	1210	1.022	*E. coli* 10^6^	Worsening ataxia, lethargy, panting, restlessness	Polyuria, hyperthermia	Improved within 24 h after amoxicillin-clavulanic acid	5
5	Kangal	fn	6.5	57.4	23.3	n/a	1.020	*E. coli* 10^6^	Restlessness, anxiety, polydipsia	Vaginal discharge, urinary incontinence, polydipsia	Improved within 48 h after amoxicillin-clavulanic acid	1
6	Australian Doodle	m	3.75	39.0	n/a	1042	1.038	*E. coli* 10^6^	Lethargy, ataxia	Polyuria, malodorous urine	Improved within 72 h after amoxicillin-clavulanic acid	2
7	Border Collie x	f	2.5	20.4	25.0	955	1.042	*E. coli* 10^6^	Lethargy, restlessness, polyuria, ataxia, weakness	Urinary incontinence, nocturia, stranguria	Improved within 48 h after amoxicillin-clavulanic acid	1
8	Greater Swiss Mountain Dog	m	2.5	47.0	19.3	n/a	1.013	*E. coli* 10^6^	Relapse of cluster seizures, ataxia, polyuria	Hyperthermia, polyuria	Improved within 48 h after amoxicillin-clavulanic acid	0

## Data Availability

The raw data supporting the conclusions of this article will be made available by the corresponding author upon request.

## Supplementary Materials

The following supporting information can be downloaded at https://www.mdpi.com/article/10.3390/ani15172562/s1. Table S1: Summary of Clinical Data for Dogs with Idiopathic Epilepsy and *E. coli* UTIs. Table S2: Phenobarbital dosages, serum concentrations, and potassium bromide dosages in eight dogs with idiopathic epilepsy at different time points before, during, and after urinary tract infection (UTI).

## Abbreviations

The following abbreviations are used in this manuscript:

IE	Idiopathic epilepsy
UTI	Urinary tract infection
ASM	Anti-seizure medication
UCG	Urinary specific gravity
*E. coli*	*Escherichia coli*
QoL	Quality of life
IVETF	International Veterinary Epilepsy Task Force
BID	Twice daily
