# Supplementary figures and images for: Clinical Deterioration in Dogs with Idiopathic Epilepsy Caused by E. coli Urinary Tract Infection
Source: Animals (Basel). 2025 Aug 31;15(17):2562. doi: 10.3390/ani15172562 (PMC12427457; doi:10.3390/ani15172562)

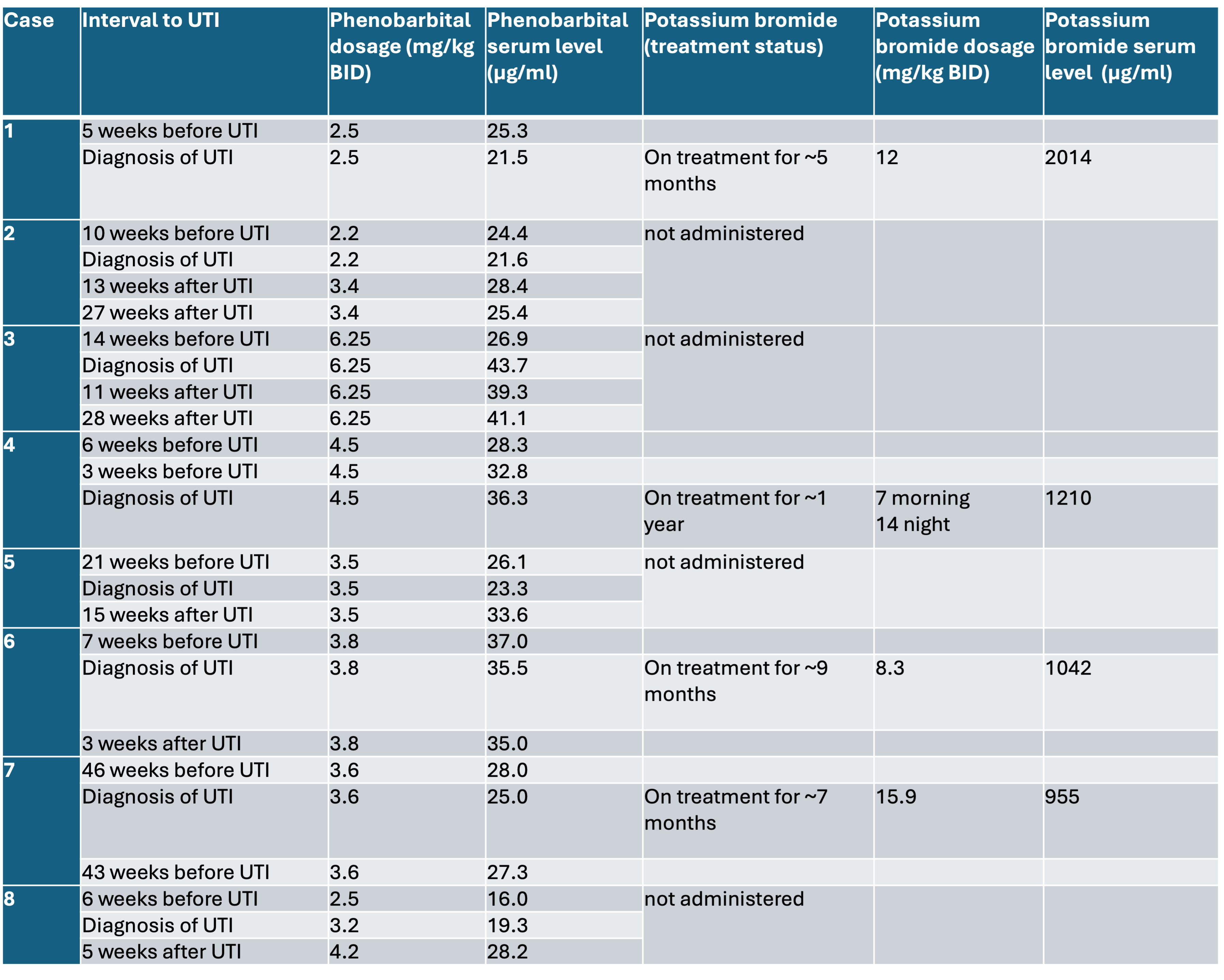

Supplement: Supplementary file 1 [file animals-15-02562-s001.zip › Supplementary Material Table S2.png]
